# Characterization of Motor-Evoked Responses Obtained with Transcutaneous Electrical Spinal Stimulation from the Lower-Limb Muscles after Stroke

**DOI:** 10.3390/brainsci11030289

**Published:** 2021-02-26

**Authors:** Yaejin Moon, Taylor Zuleger, Martina Lamberti, Ashir Bansal, Chaithanya K. Mummidisetty, Kelly A. McKenzie, Lindsey Yingling, Sangeetha Madhavan, Elliot J. Roth, Richard L. Lieber, Arun Jayaraman

**Affiliations:** 1 Shirley Ryan AbilityLab, Chicago, IL 60611, USA; ymoon@sralab.org (Y.M.); cmummidise@sralab.org (C.K.M.); kmckenzie@sralab.org (K.A.M.); lkyingling@gmail.com (L.Y.); eroth@sralab.org (E.J.R.); rlieber@sralab.org (R.L.L.); 2 Feinberg School of Medicine, Northwestern University, Chicago, IL 60611, USA; ashir.bansal@northwestern.edu; 3 Department of Neuroscience, University of Cincinnati, Cincinnati, OH 45221, USA; zulegetm@mail.uc.edu; 4 Clinical Neurophysiology, University of Twente, 7522 NH Enschede, The Netherlands; m.lamberti@utwente.nl; 5 Department of Physical Therapy, University of Illinois, Chicago, IL 60612, USA; smadhava@uic.edu; 6 Edward Hines Jr. V.A. Hospital, Maywood, IL 60141, USA

**Keywords:** stroke, spinal cord, electrical spinal cord stimulation, spinal motor-evoked response

## Abstract

An increasing number of studies suggests that a novel neuromodulation technique targeting the spinal circuitry enhances gait rehabilitation, but research on its application to stroke survivors is limited. Therefore, we investigated the characteristics of spinal motor-evoked responses (sMERs) from lower-limb muscles obtained by transcutaneous spinal cord stimulation (tSCS) after stroke compared to age-matched and younger controls without stroke. Thirty participants (ten stroke survivors, ten age-matched controls, and ten younger controls) completed the study. By using tSCS applied between the L1 and L2 vertebral levels, we compared sMER characteristics (resting motor threshold (RMT), slope of the recruitment curve, and latency) of the tibialis anterior (TA) and medial gastrocnemius (MG) muscles among groups. A single pulse of stimulation was delivered in 5 mA increments, increasing from 5 mA to 250 mA or until the subjects reached their maximum tolerance. The stroke group had an increased RMT (27–51%) compared to both age-matched (TA: *p* = 0.032; MG: *p* = 0.005) and younger controls (TA: *p <* 0.001; MG: *p* < 0.001). For the TA muscle, the paretic side demonstrated a 13% increased latency compared to the non-paretic side in the stroke group (*p* = 0.010). Age-matched controls also exhibited an increased RMT compared to younger controls (TA: *p* = 0.002; MG: *p* = 0.007), suggesting that altered sMER characteristics present in stroke survivors may result from both stroke and normal aging. This observation may provide implications for altered spinal motor output after stroke and demonstrates the feasibility of using sMER characteristics as an assessment after stroke.

## 1. Introduction

A growing number of studies support a novel neuromodulation technique that targets the lumbosacral spinal circuitry and enhances gait function after spinal cord injury (SCI). Specifically, epidural and non-invasive continuous electrical spinal stimulation techniques aim to harness spared and silent descending pathways within the spinal circuitry after SCI [1]. Preclinical and clinical studies have shown that continuous spinal stimulation can elicit tonic and rhythmic patterns of motor activity during walking or step-like movements, despite limited communication with the brain in individuals with SCI [1]. These observations highlight the critical role of spinal networks in processing and dynamically modulating sensory input to generate efferent activity appropriate to the phase and task of walking [2,3]. Based on these exciting results, it has been proposed that continuous transcutaneous spinal cord stimulation (tSCS) can provide a simple, safe, and non-invasive approach to treating a wide range of neurological diseases with gait disorders, including stroke [4]. However, to the best of our knowledge, no studies have applied spinal cord stimulation to stroke survivors.

A fundamental parameter to be explored, prior to using continuous tSCS as a neuromodulation technique in stroke, is eliciting spinal motor-evoked responses (sMERs) in the population. An sMER examines the muscle response evoked by a single pulse of tSCS to investigate surrogate electrophysiological changes in the spinal neuronal networks. Previous studies have referred to the muscle responses evoked by this method as multisegmental monosynaptic responses (MMRs) [5], posterior root-muscle reflex (PRM) [6], spinally evoked motor potentials (sEMPs) [2], or trans-spinal evoked potentials (TEPs) [7,8]. An sMER is considered the basic component of lower-limb muscle responses elicited by electrical stimulation of posterior lumbar cord structures [2,6,9,10]. In individuals without neurological injury, an sMER has been considered an analogue of Hoffmann (H)-reflex tests, since soleus H-reflex-like responses can be evoked by tSCS applied to the lumbar area [11]. However, a distinguishable characteristic is that an sMER primarily recruits dorsal root afferent fibers at proximal sites adjacent to the lumbar cord [2,6,9,10], while the H-reflex is elicited by stimulation of large-diameter afferents in the peripheral nerves [6,12,13].

Importantly, an sMER possesses distinct patterns in both motor recruitment curves and motor latencies as indirect measures of spinal representations of motor output [2,5,6,7,8,14,15,16,17,18]. Specifically, the resting motor threshold (RMT), which is the lowest stimulation intensity of tSCS eliciting muscle responses, reflects the central state of excitability of the spinal cord [7]. The slope of the recruitment curve of sMERs shows motoneuronal gain to produce a synchronized depolarization, and the latency of sMERs provides information about the spinal-to-muscle conduction time [8]. In individuals with SCI, the sMER is characterized by an increased RMT and a reduced amplitude of muscle activation, reflecting reduced spinal motor output but with preserved onset latency compared to controls without SCI [7,19]. These results have directed therapeutic programs using continuous tSCS in the SCI population to upregulate spinal excitability over multiple segments by bringing motoneurons closer to the threshold with the stimulation [7,17].

To explore whether tSCS approaches could be meaningful to stroke survivors, we identified the characteristics of sMERs obtained with a single pulse of tSCS from the lower-limb muscles after stroke and compared these findings to both age-matched and younger control groups. Even though stroke has a cortical etiology, several studies have reported pathological changes in the spinal cord after stroke by examining animal models, neuroimaging techniques, or H-reflex tests. Specifically, Dang et al. (2016) reported that the number of disordered myelin sheaths significantly increased in the lumbar spinal ventral root in rats after inducing stroke [20]. In addition, a study that examined the spinal cord using magnetic resonance imaging in stroke survivors observed morphological changes in sensorimotor pathways in the spinal cord and decreased ipsi-lesional corticospinal tract integrity [21]. Lastly, H-reflex studies have reported reduced spinal presynaptic inhibition, which could potentially contribute to spasticity after stroke [22,23,24]. Therefore, we hypothesized that the individuals with stroke would demonstrate altered spinal motor responsiveness in sMER characteristics compared to controls and also show measurable differences between the paretic side and the non-paretic side. We also hypothesized that the age-matched control group would exhibit a different spinal motor output compared to younger participants due to natural neuronal degeneration associated with aging [25].

## 2. Materials and Methods

### 2.1. Participants

Ten stroke survivors, ten age-matched controls without stroke, and ten younger controls were recruited for this study. Stroke survivors were recruited from a research volunteer registry and the local community. Control group participants were recruited through digital advertisements placed in the local community. Inclusion criteria for the stroke group were as follows: (1) age 18 years or more, (2) at least 6 months’ post-stroke, (3) hemiparesis/hemiplegia after a single stroke, and (4) functional ambulation category of 2 or greater. Exclusion criteria for the stroke group were as follows: (1) currently receiving regular physical therapy services, (2) currently taking a selective serotonin reuptake inhibitor or tricyclic antidepressant medication, (3) botulinum toxin injection in the lower limb within the prior 4 months, (4) modified Ashworth scale (MAS) of 3 or greater in the lower extremity, (5) presence of a pacemaker or implantable intrathecal pump, (6) presence of painful musculoskeletal dysfunction, (7) history of seizures, (8) presence of cardiac arrhythmias, and (9) metal implants in the spine or back.

Age-matched and younger control participants were included if they had no known history of stroke or neurological degenerative pathologies, no metal implants in the spine or back, and no implanted cardiac device. Age-matched controls were between the ages of 45 and 75 years, and younger control participants were between the ages of 18 and 30 years. All participants provided informed consent before they participated in the study. All study-related procedures were approved by the Northwestern University Institutional Review Board (NUIRB, STU00206430-MOD0024), Northwestern University, Chicago, IL, USA.

Prior to the spinal stimulation experiment, participants in the stroke group underwent functional examination, including the lower-extremity Fugl–Meyer (FMA-LE) and modified Ashworth scale (MAS) measures.

### 2.2. Transcutaneous Electrical Spinal Cord Stimulation (tSCS) Procedure

**Figure 1:**
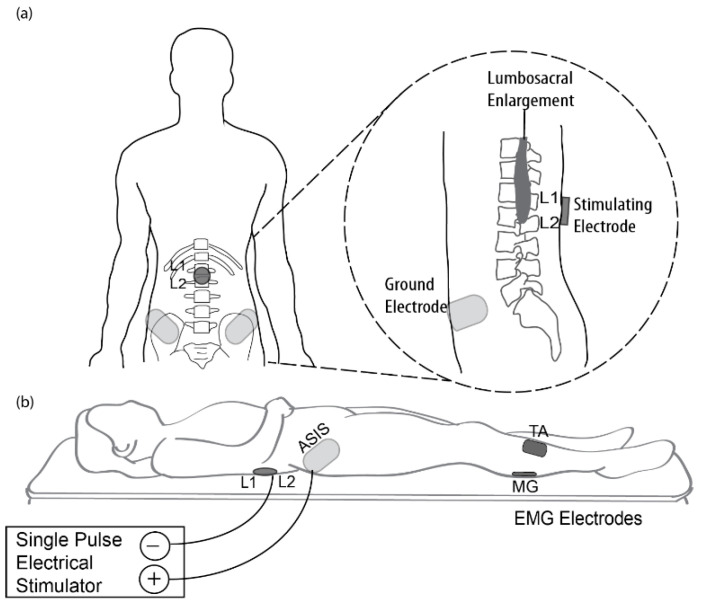
Transcutaneous electrical spinal cord stimulation (tSCS) setup. (**a**) Sketch of the placement of stimulating and reference electrodes between L1 and L2 spinous processes and the anterior superior iliac spine (ASIS), respectively, relative to the lumbosacral enlargement. (**b**) Experiment setup of single-pulse tSCS showing the posture of the participants, positions of anode and cathode electrodes, and electromyographic (EMG) recordings from tibialis anterior (TA) and medial gastrocnemius (MG) muscles.

Figure 1 illustrates the tSCS experimental setup. The L1 spinous process was identified via palpation by an experienced physical therapist. A single cathode electrode (ValuTrode, Axelgaard Ltd., Fallbrook, CA, USA) with a diameter of 3.2 cm was placed medially between the L1 and L2 spinous processes (Figure 1a). The L1–L2 vertebral levels were selected because they correspond to L5–S5 spinal segments, which is the location of the lumbosacral enlargement of the spinal cord that innervates the tibialis anterior (TA) and medial gastrocnemius (MG) muscles [26,27]. Additionally, a pair of anode electrodes (UltraSim, Axelgaard Ltd., 7.5 × 13 cm, Fallbbrook, CA, USA) were placed symmetrically over the anterior superior iliac spines (ASIS). Cathode and anode electrodes were connected to a custom-built constant current stimulator (BioStim-5, Cosyma, Moscow, Russia) [28], which was used to deliver single monophasic rectangular-wave pulses with a pulse width of 1 ms. After electrode placement, participants were asked to lie in a relaxed, supine position. Participants were guided to maintain a stable position and to avoid moving the limbs during testing. Stimulation was delivered at 5 mA increments, increasing from 5 mA to 250 mA or until the subject reached maximum tolerance. Each stimulation intensity was delivered three times [2,5,6,14], in 5 s intervals [27]. The sMER resulting from each stimulation pulse was recorded. In addition, the maximum intensity of tSCS that was tolerated by each participant was documented.

### 2.3. Electromyographic (EMG) Recordings

Surface electromyographic (EMG) activity of sMERs was recorded with bipolar Ag-AgCl surface electrodes (GS26, Bio-Medical Instruments, Clinton Charter Township, MI, USA). Each electrode pair was placed longitudinally on the belly of the TA and MG muscles. Recordings from the TA and MG muscles were selected, as it has been reported that distal muscles have greater motor deficits compared to more proximal muscles in stroke survivors [29]. Ground reference electrodes were placed bilaterally over the bony prominence of the patella (Figure 1b). EMG signals were sampled at 4000 Hz and bandpass-filtered (fourth-order Bessel filter, 30–2000 Hz) by the PowerLab 16/35 data acquisition system operated with LabChart software (v7.2, AD Instruments, Bella Vista, NSW, Australia).

### 2.4. Data Analysis

**Figure 2:**
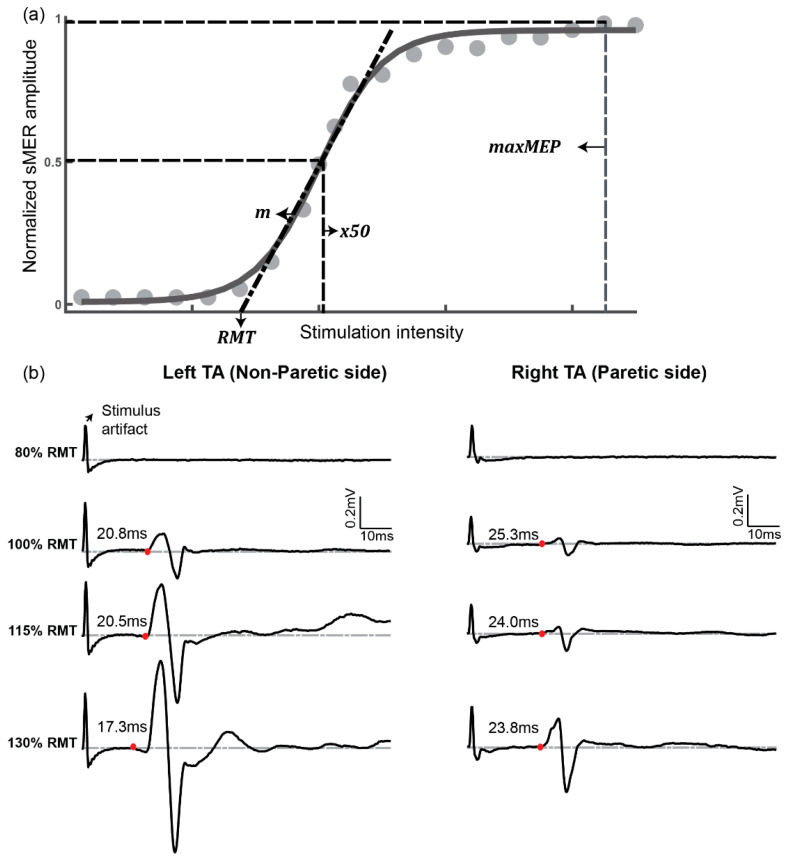
Representative spinal motor-evoked responses (sMERs). (**a**) The sMER recruitment curve for a single subject, based on peak-to-peak amplitude of averaged MER at each stimulation intensity. The points were fitted by a sigmoid function. Drop lines indicate the stimulator output at the resting motor threshold (RMT), x50, which is the stimulus intensity required to obtain an MER that is 50% of the maximum. (**b**) sMERs recorded across a range of responses evoked at different stimulation intensities expressed as a percentage of the RMT of a single subject. Latency was calculated as the time from the stimulation delivery to the first deflection of sMER data (red dot).

Three electrophysiological parameters were measured to characterize the responsiveness of lower-limb muscles to tSCS: resting motor threshold (RMT), slope of the sMER recruitment curve, and latency. The detailed significance of the outcome parameters are described in Table 1. The RMT and slope were calculated based on the sMER recruitment curve. To generate recruitment curves, the peak-to-peak amplitude was calculated as a representative of the sMER size of each stimulation trial. For each intensity, sMER sizes were averaged across three sets of stimulation trials. Prior to further analysis, the averaged sMER size at each intensity was normalized to the maximal sMER size of the corresponding muscle [6,7,8,15]. Finally, sMER recruitment curves were plotted as stimulation intensities against the normalized sMER size (see the sample in Figure 2a).

To quantify the RMT and slope, the sMER recruitment plot was fitted to a sigmoid function using a custom script in MATLAB (Mathworks, Inc., Natick, MA, USA) [15,31]. The following four parameters were derived from the coefficients of the sigmoid fitting function (Equation (1)): (1) *minMER*, defined as the minimum value of the data; (2) *maxMER*, defined as the maximum value of the data; (3) *x50,* defined as the stimulation level corresponding to the sMER at 50% of *maxMER*; and (4) slope parameter *m,* defined as the derivative of the sMER recruitment curve calculated at *x50*. After applying the fitting function to the data, the slope of the sMER recruitment curve was obtained by calculating the derivative of the sigmoid function at *x50*. The RMT was defined as the intercept on the *x* axis of the linear extrapolation of the line tangential to the sMER recruitment curve at *x*50.

(1) $$S\left(x\right)=minMER+\frac{maxMER-m}{1+exp\left(m\left(x50-x\right)\right)}$$

Additionally, sMER latency recorded at the intensity from 100% to 130% RMT was estimated, since all participants tolerated stimulation over this intensity range. The latency for each muscle group was determined using the raw EMG data from each stimulation trial (Figure 2b). Latency was defined as the time from stimulation delivery to the first positive or negative deflection of EMG data from the baseline. The deflection point of EMG data was defined as when the averages of a six-bin window of EMG values and the following five windows were each greater than two standard deviations from the baseline average [32].

### 2.5. Statistical Analysis

**Figure 3:**
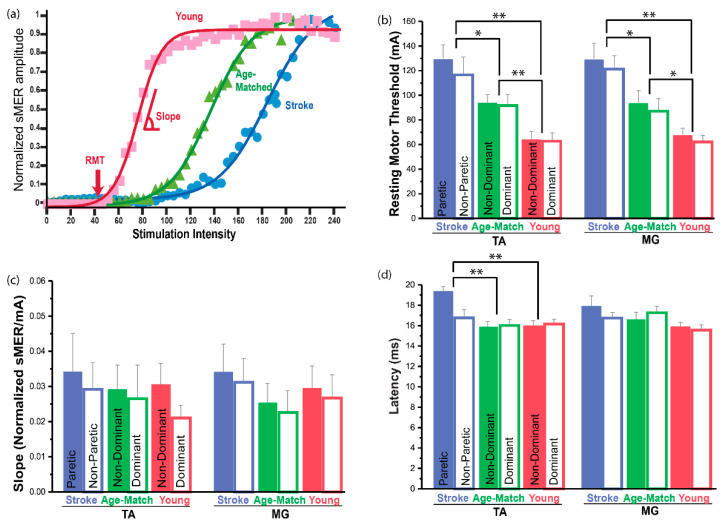
Comparison of electrophysiological parameters of sMERs by group and side. (**a**) sMER recruitment curves of a representative participant from each group. The recruitment curve of the age-matched control group shifted to the right (i.e., higher RMT) compared to the younger control group, while that of the stroke group further shifted to the right compared to the age-matched control group. These participants had similar slopes, but the RMT was the highest for the stroke group, followed by the age-matched control group, with the younger control group having the lowest RMT. Electrophysiological parameters presented for TA and MG muscles as a function of group and side. (**b**) Resting motor threshold (RMT), (**c**) slope of sMER recruitment curve, and (**d**) sMER latency. Data are presented as the mean ± standard error (SE) * *p* < 0.050; ** *p* < 0.010.

Statistical analysis was performed using SPSS for Windows, version 26.0 (IBM, Inc., Chicago, IL, USA). Normality of outcome measures was tested using the Shapiro–Wilk test. When the normality assumption was violated, data were log-transformed. For categorical demographic data, the Pearson chi-square test was used for group comparisons. Continuous demographic data were compared among groups by analysis of variance (ANOVA). To compare the maximum tolerated intensity of tSCS among groups, the Kruskal–Wallis H test was used. The intrasubject variability of responses to single-pulse tSCS (peak-to-peak amplitude and latency) was computed as a coefficient of variation (CV = 100 × standard deviation/mean) of the three trials at each stimulation intensity. To examine the group effect on sMER parameters (threshold, slope, and latency), one-way ANOVA was conducted with group (stroke, age-matched, or younger) as the factor. When a significant group effect was observed, post hoc paired comparisons were conducted with Fisher’s LSD test. A measure of eta-squared (*η^2^*) was obtained as the effect size for one-way ANOVA. Conventionally, *η^2^* values of 0.01, 0.06, and 0.14 are considered to represent small, medium, and large effect sizes, respectively [33]. Since both age-matched and younger control groups showed no significant difference between the dominant and non-dominant side for all sMER parameters (all *p* > 0.050; see Figure 3), only sMER data recorded from the non-dominant side of the controls were considered. To examine the effect of side (paretic vs. non-paretic) within the stroke group on sMER parameters, a paired *t*-test was computed. For the paired *t*-test, the effect size of Cohen’s *d* was calculated, with the values of 0.2, 0.5, and 0.8 interpreted as small, medium, and large effects, respectively. All tests were performed with a two-sided test. *p*-Values equal to or less than 0.05 were considered statistically significant.

## 3. Results

### 3.1. Demographics

There was no significant age difference between the stroke and the age-matched control group (*U* = 45.0, *p* = 0.739), while the younger control group was significantly younger than both stroke (*p* < 0.001) and age-matched control groups (*p* < 0.001; Table 2). There was a greater proportion of males in the stroke group (70%) compared to the age-matched (20%) and the younger (30%) group, but the difference did not reach a statistically significant level (*x*^2^_(2,__*n*__=30)_ = 5.83, *p* = 0.053). There was no significant group effect in height (F_(2,27)_ = 2.00, *p* = 0.155), body mass index (BMI) (F_(2,27)_ = 3.03, *p* = 0.065), and handedness (*x*^2^_(2,__*n*__=30)_ = 2.22, *p* = 0.329). Three participants (30%) in the stroke group were impaired on their dominant side. The average Fugl–Meyer lower-extremity score for the stroke group was 22.2 ± 3.9 (range: 14–26).

### 3.2. Participants’ Tolerance to a Single Pulse of tSCS

To monitor the participants’ safety during sMER testing, we documented the maximum intensity of a single pulse of tSCS tolerated by each participant. Each time, prior to proceeding to the next level of stimulation intensity, participants were asked whether there was any discomfort or pain and whether they were comfortable trying the next stimulation level. Of 30 participants, 17 voluntarily stopped before reaching the maximum stimulation intensity (i.e., 250 mA). On average, the stroke group tolerated single-pulse tSCS up to 202.5 ± 11.6 mA (median: 195 mA, Interquartile range (IQR): 186.25–230 mA), whereas the age-matched control group tolerated it up to 209.0 ± 17.0 mA (median: 235 mA, IQR: 186.25–250 mA), and the younger control group tolerated it up to 218.5 ± 13.5 mA (median: 250 mA, IQR: 183.75–250 mA). There was no significant difference in these maximum tolerated intensities among the groups (H_(2)_ = 1.122, *p* = 0.571). Follow-up phone calls/emails confirmed that none of the participants experienced self-reported pain or discomfort during or following the experiment.

### 3.3. Variability of Responses to Single-Pulse tSCS

Intrasubject variability of responses to a single pulse of tSCS was computed by the CV of three trials at each stimulation intensity. The average CV of the peak-to-peak amplitude of sMERs was 9.2% ± 7.6% for the stroke group, 10.9% ± 5.1% for the age-matched control group, and 10.9% ± 4.6% for the younger control group. The average CV of the onset latency of sMERs was 3.9% ± 2.9% for the stroke group, 4.3% ± 3.9% for the age-matched control group, and 4.5% ± 3.8% for the younger control group. There was no significant group difference in the variability of sMERs for both peak-to-peak amplitude (H_(2)_ = 2.282, *p* = 0.320) and latency (H_(2)_ = 1.178, *p* = 0.528).

### 3.4. Resting Motor Threshold (RMT)

The RMT was the highest for the stroke group, followed by the age-matched and younger control groups (Figure 3a,b and Table S1). Statistical analyses demonstrated that there was a significant group effect in both muscles (TA: F_(2,27)_ = 13.73, *p* < 0.001, *η^2^* = 0.50; MG: F_(2,27)_ = 9.89, *p* = 0.001, *η^2^* = 0.42). Post hoc analysis revealed that the stroke group had a significantly greater RMT compared to the age-matched control group (TA: *p* = 0.041; MG: *p* = 0.032) and the younger control group for both muscles (TA: *p* < 0.001; MG: *p* < 0.001). When compared to the age-matched control group, the stroke group had a 27% and 28% higher RMT in TA and MG muscles, respectively. When compared to the younger control group, the stroke group had a 51% and 48% higher RMT in TA and MG muscles, respectively. Additionally, post hoc analyses indicated that the age-matched control group had a significantly greater threshold compared to the younger control group for both muscles (TA: *p* = 0.005, 32% greater; MG: *p* = 0.038, 28% greater). There was no significant side effect (paretic vs. non-paretic) within the stroke group in the RMT for either muscle (TA: t_(9)_ = 1.12, *p* = 0.293; MG: t_(9)_ = 0.57, *p* = 0.585). Overall, our results demonstrated that both stroke and aging contribute to increased RMT, which results in reduced spinal motor responsiveness of the lower-limb muscles.

### 3.5. Slope of the sMER Recruitment Curve

Statistical analyses revealed no significant difference of slope among groups for either muscle (TA: F_(2,27)_ = 0.09, *p* = 0.910; MG: F_(2,27)_ = 0.34, *p* = 0.718; Figure 3c, Table S2). Additionally, no effect of side was found for the slope of curves from either muscle (TA: t_(9)_ = 0.23, *p* = 0.827; MG: t_(9)_ = 0.15, *p* = 0.882). This indicated that neither aging nor the stroke side influences the rate of recruiting additional sensory and motor neurons with increasing stimulation intensity.

### 3.6. Latency

A significant group effect was observed for sMEP latency for the TA muscle (F_(2,27)_ = 14.44, *p* < 0.001, *η^2^* = 0.52; Figure 3d and Table S3). Post hoc analyses revealed that for the TA muscle, the stroke group exhibited significantly increased latency compared to both control groups (age-matched: *p* < 0.001, 17% greater; younger: *p* < 0.001, 17% greater). Additionally, for the TA muscle, the paretic side demonstrated significantly increased latency compared to the non-paretic side (t_(9)_ = 2.82, *p* = 0.20, d = 0.35, 13% increase). However, similar group or side effects in latency were not observed for the MG muscle. These results demonstrated that stroke contributes to delayed latency, especially in the TA muscle, reflecting increased signal propagation time from the spinal nervous system to the peripheral musculature on the paretic side.

## 4. Discussion

This study identified the characteristics of spinal motor-evoked responses obtained with tSCS from lower-limb muscles of stroke survivors compared to age-matched and younger controls. The principal findings were that (1) the stroke group exhibited an increased RMT and increased latency compared to both age-matched and younger control groups, (2) the TA muscle on the paretic side had increased latency compared to the non-paretic side, (3) there was no significant difference in the slope of the sMER recruitment curve among the groups, and (4) the normal aging process is also associated with an increased RMT.

### 4.1. Changes in the Spinal Motor Responsiveness after Stroke

The current study observed reduced spinal motor responsiveness after stroke, reflected by an increased RMT (TA: 27% increase, MG: 28% increase) and delayed TA latency (13% increase) in stroke subjects compared to the age-matched control group. This suggests that cortical damage after stroke might downregulate spinal motor responsiveness to the lower-limb muscles. Studies describing transcranial magnetic stimulation (TMS) applied to the cortex have reported an increased RMT and increased latency for people with stroke compared to controls [34,35]. Thus, keeping in mind the current study, it is interesting to observe the similarity in impaired motor responsiveness at the cortical and spinal cord levels, leading us to attribute the secondary changes in the spinal cord circuitry to the primary cortical lesion.

In contrast, studies that have examined H-reflex changes after stroke have reported hyper-excitable spinal reflexes demonstrating a lower RMT and decreased latency in stroke survivors compared to those without stroke [22,23]. H-reflex studies in individuals with a cortical lesion showed reduced spinal presynaptic inhibition, which could contribute to the hyper-excitability of the H-reflex [24]. It is unclear what causes the discrepancy between the current observation of sMERs (i.e., reduced spinal motor output) and H-reflex literature (i.e., hyper-excitable spinal reflex) in stroke survivors. One reason could be the differences in involved neural pathways during testing. Specifically, whereas the H-reflex is elicited by stimulating large-diameter afferents in the peripheral nerves [6,12,13], the sMER is evoked primarily in the dorsal root afferent fibers [2,6,9,10] and/or many other neural structures (e.g., synapses, neuronal cell bodies, and glial cells) at proximal sites adjacent to the lumbosacral region of the spinal cord impacted by the electrical field [1].

Furthermore, there are different approaches to tSCS. A single-pulse tSCS protocol has been considered to reflect changes in multisegmental spinal motoneuron output [2,5,6,7,8,14,15,16,17,18], while a paired-pulse tSCS protocol was established to examine post-activation inhibitory effects [5,6,7,15,27]. Therefore, it would be interesting to compare the current results to a paired-pulse tSCS protocol in stroke survivors.

It is also important to note that both paretic and non-paretic sides exhibited an increased RMT. This observation was in line with previous studies that suggested that changes in cortical excitability after stroke appear to occur bilaterally [36]. Potentially, alteration of corticospinal input might affect the overall responsiveness of the lumbosacral spinal network bilaterally. An alternative explanation could be that overall disuse of lower-limb muscles due to decreased functional activity has a retrograde influence on spinal networks underlying motor function [37], but this idea was not experimentally tested in this study.

Another interesting observation was that compared to the controls, only the TA muscle exhibited significantly increased latency on the paretic side, while the MG muscle did not. This observation may be explained by the fact that different neural pathways control TA and MG muscles. TMS studies reported that there are stronger connections between corticospinal tract fibers and distal leg flexors (e.g., TA muscle) than to the extensors (e.g., MG muscle), and thus corticospinal tract fibers disrupted by stroke could result in greater impairment in the TA muscle compared to the MG muscle [38,39]. The current study also observed different deficits between the dorsiflexor (TA) and plantar flexor (MG) of the stroke group. Specifically, the dorsiflexors had lower levels of active motion (FMA-LE subscore (max = 2): 0.8 ± 0.3) compared to the plantar flexors (FMA-LE subscore: 1.3 ± 0.2). 

### 4.2. No Change in the Slope of the sMER Recruiment Curve after Stroke

There was neither a group nor a side effect for the slope of the sMER recruitment curve. Although the RMT increases after stroke, the ability to recruit additional sensory and motor neurons in an orderly fashion with increasing stimulation intensity appears to remain intact. It has been suggested that electrical stimulation recruits larger-diameter axons prior to smaller-diameter axons, since larger neurons have a lower resistance and conduct action potentials at faster rates compared to neurons with smaller axons [40,41]. Therefore, it is possible that despite the increased RMT after stroke, the recruitment order, which is based on the biophysical properties of axons, does not change. 

However, it should also be noted that the stroke group had greater inter-subject variability (standard error (SE) = 0.011) in the slope of the recruitment curve compared to both control groups (age-matched: SE = 0.007; younger: SE = 0.006). Therefore, caution is needed when generalizing the influence of stroke on the slope of the recruitment curve. The statistical power for the current results of slope was 68% (effect size f = 0.45, correlation among repeated measures = 0.46). A post hoc calculation showed that a total of 60 participants (20 participants in each group) are needed to provide 95% power at the 5% level of significance. Therefore, a future study with a larger sample size can clarify these findings.

### 4.3. Effects of Aging

Another interesting finding of this study was that the age-matched control subjects exhibited a significant increase in the RMT (28–32% increase) compared to the younger control subjects. Prior studies have also reported age-related changes in cortical and spinal pathways [25,42]. Specifically, it was reported that approximately 35% of corticospinal motoneurons are either lost or become non-functional in normal humans by the age of 50 years [43]. This observation is consistent with our observations, considering the average age of the age-matched control group was 55.5 ± 2.6 years. Therefore, it is likely that there was a cumulative effect on changes in sMER characteristics in stroke subjects due to both stroke and normal aging. 

### 4.4. Clinical Implication

Reduced spinal motor responsiveness after stroke supports the concept that priming lumbar cord networks could be another/additional approach to maximizing neural recovery in stroke survivors. In SCI studies, applying low-frequency (0.2 Hz) continuous tSCS altered the central state of excitability of lumbar cord networks over multiple segments by bringing motoneurons closer to the threshold, making them more easily depolarized to descending and local inputs [7]. Additionally, a combination of 30 Hz continuous tSCS and gait training restored voluntary rhythmic leg movement in people with chronic complete SCI [17]. One caveat of upregulating the spinal circuit using tSCS is that it might deteriorate spasticity, as stroke survivors exhibit a hyper-excitable spinal reflex [24]. However, interestingly, a previous study with incomplete SCI showed significant suppression of severe lower limb spasticity when 50 Hz continuous tSCS was placed over lumbar posterior roots [44]. It was speculated that tSCS depolarized posterior root fibers to make stronger synaptic connections to Ia inhibitory interneurons [44]. It might seem contradictory that tSCS can both upregulate the motor response and also downregulate the spinal reflex. However, this observation was in line with a report from Murray et al. (2018), who observed that continuous direct current spinal stimulation increased facilitatory mechanisms on corticospinal excitability, whereas it reduced soleus H-reflex excitability [18]. Since our study suggests that there are changes in motor responsiveness within the lumbar cord after stroke, it may be promising to apply continuous tSCS to enhance lower-limb function in individuals who have experienced a stroke. 

### 4.5. Limitations

There were several limitations of this study. The major limitation was the small sample size, which might have resulted in failure to detect significant group differences. Another notable limitation was that the both control groups had a greater number of female participants (age-matched: 80% female, younger: 70% female), while the stroke group was predominantly male (70% male). Although prior studies on MERs elicited by TMS reported no gender differences in cortical excitability [45], it would be valuable to examine the effect of gender on the spinally evoked motor response. Further, the stroke participants in this study had several unique characteristics (e.g., chronic stroke, mainly male gender, primary impairment on the non-dominant side, minimum spasticity) that may also limit the generalizability of our results. Additionally, variability in the anatomical arrangement of the spinal cord and vertebral column, as well as the amount of subcutaneous fat, skin resistance, muscle density, and intervertebral ligament structure, must be taken into account. While we did not find statistical significance in the BMI of the participants, potential confounding factors should be considered when evaluating the current data. Finally, although it is a common practice to place the electrodes on the designated vertebrae level with palpation for tSCS application [7,8,11,15,16,44], it is possible that the electrode location was not potentially optimal.

## 5. Conclusions

The present results are the first that we know of that provide evidence of secondary changes in the downstream lumbar spinal neural networks and descending pathways after stroke in human subjects. This observation may provide additional insight into pathological mechanisms that influence lower-limb motor impairment following stroke. Additionally, the present results suggest that the spinal neural network can be a novel target for neuromodulation of movement rehabilitation of stroke survivors. Further studies are needed that explore the mechanisms and implications of spinal stimulation for neural recovery and function.

## Figures and Tables

**Table 1:** Significance of outcome measures.

Outcome Measures	Definition	Physiological Significance of Change in Metric
Resting motor threshold (RMT)	Stimulation intensity required to activate the most excitable spinal motor pools without any voluntary muscle activation	An increased RMT indicates decreased ion channel conductivity and hence decreased membrane excitability of neurons in the spinal motor pools [30].
Slope of sMER recruitment curve	Rate at which sMER size increases with increasing stimulus intensity	A decreased slope represents a decreased rate to recruit additional motor neurons with increasing stimulation intensity [2].
Latency	Signal propagation time between stimulation of the spinal cord to the onset of muscle response	Increased latency indicates delayed conduction time in axons originating from the spinal cord [7].

**Table 2:** Demographic information about study participants.

	Stroke	Age-Matched	Younger
*n*	10	10	10
Gender	7 M: 3 F	2 M: 8 F	3 M: 7 F
Age (years)	56.8 ± 2.3	55.5 ± 2.6	24.8 ± 0.9
Height (cm)	172.6 ± 1.9	166.3 ± 2.2	172.0 ± 3.1
BMI (kg/m^2^)	28.9 ± 1.8	25.3 ± 1.2	23.7 ± 1.4
Handedness	10 R: 0 L	9 R: 1 L	8 R: 2 L
Paretic side	3 R: 7 L	--	--
Time since stroke (years)	5.9 ± 1.1	--	--
Type of stroke	4 Hem: 6 Isc		
FMA-LE	22.2 ± 3.9		
MAS (dorsiflexor)	All scored 0		
MAS (plantar flexor)	1.0 ± 0.2		

Note: M, Male; F, Female; BMI, body mass index; R, Right; L, Left; Hem, hemorrhagic stroke; Isc, ischemic stroke; FMA-LE, Fugl–Meyer lower extremity; MAS, modified Ashworth scale. Data are presented as the mean ± standard error (SE).

## Data Availability

The datasets generated and/or analyzed during the current study are available from the corresponding author on reasonable request.

## Supplementary Materials

The following are available online at https://www.mdpi.com/2076-3425/11/3/289/s1: Table S1: Statistical results of resting motor threshold as a function of group and side; Table S2: Statistical results of slope of the sMER recruitment curve as a function of group and side; Table S3: Statistical result of onset latency as a function of group and side.
