# Supplementary material for: Characterization of Motor-Evoked Responses Obtained with Transcutaneous Electrical Spinal Stimulation from the Lower-Limb Muscles after Stroke
Source: Brain Sci. 2021 Feb 26;11(3):289. doi: 10.3390/brainsci11030289 (PMC7996860; doi:10.3390/brainsci11030289)
Supplement: Supplementary file 1 [file brainsci-11-00289-s001.pdf]

Supplementary Materials

Characterization of motor evoked responses obtained with transcutaneous electrical spinal stimulation from the lower-limb muscles post stroke

Yaejin Moon, PhD,*[1,2] Taylor Zuleger, BS,*[3] Martina Lamberti, MS,*[4] Ashir Bansal, BS,[2] Chaithanya K. Mummidisetty, MS,[1] Kelly A. McKenzie, DPT,[1] Lindsey Yingling, DPT,[1] Sangeetha Madhavan, PT, PhD,[5] Elliot J. Roth, MD,[1,2] Richard L. Lieber, PhD,[1,2,6] Arun Jayaraman, PT, PhD[1,2]

[1] Shirley Ryan AbilityLab, Chicago, IL 60611, USA

[2]Feinberg School of Medicine, Northwestern University, Chicago, IL 60611, USA

[3]Department of Neuroscience, University of Cincinnati, Cincinnati, OH, 45221, USA

[4]Clinical Neurophysiology, University of Twente, Enschede, The Netherlands

[5]Department of Physical Therapy, University of Illinois, 1919 W Taylor St, Chicago, IL 60612, USA

[6]Edward Hines Jr, V.A. Hospital, Maywood IL 60141, USA

S1 Table: Statistical results of resting motor threshold as a function of group and side.

Resting Motor Threshold (mA)		Paretic (Non-Dominant)		Non-Paretic		Group effect	Group Difference post-hoc analysis	Side effect
		Mean	S.E.	Mean	S.E.			
TA	Stroke	128.98	10.83	116.52	13.61	$F_{(2,27)}$=13.73, p<.001*, η^2=.50	Stroke vs. Age-Matched: p=0.041* Stroke vs. Young: p<0.001* Age-Matched vs. Young: p=0.005*	$t_{(9)}$=1.12, p=.293
	Age-Matched	93.56	6.50	--	--			
	Young	63.71	6.89	--	--			
MG	Stroke	128.68	12.52	121.25	9.85	$F_{(2,27)}$=9.89, p=.001*, η^2=.42	Stroke vs. Age-Matched: p=0.032* Stroke vs. Young: p<0.001* Age-Matched vs. Young: p=0.038*	$t_{(9)}$=0.57, p=.585
	Age-Matched	93.18	9.53	--	--			
	Young	67.19	4.98	--	--			

Note: For group comparison, sMEP data of the paretic-side of the stroke group were compared with that of the non-dominant side of the healthy controls. Note: *= *p*-value less than 0.050. TA=Tibialis Anterior; MG=Medial Gastrocnemius

S2 Table: Statistical results of slope of the sMER recruitment curve as a function of group and side.

Slope (normalized EMG/mA)		Paretic (Non-Dominant)		Non-Paretic		Group effect	Group Difference post-hoc analysis	Side effect
		Mean	S.E.	Mean	S.E.			
TA	Stroke	0.0341	0.0110	0.0292	0.0075	$F_{(2,27)}$=0.09, *p*=.910	N/A	$t_{(9)}$=0.23, *p*=.827
	Age-Matched	0.0291	0.0071	--	--			
	Young	0.0305	0.0060	--	--			
MG	Stroke	0.0340	0.0080	0.0312	0.0067	$F_{(2,27)}$=0.34, *p*=.718	N/A	$t_{(9)}$=0.15, *p*=.882
	Age-Matched	0.0252	0.0057	--	--			
	Young	0.0294	0.0064	--	--			

Note: For group comparison, sMEP data of the paretic-side of the stroke group were compared with that of the non-dominant side of the healthy controls. Note: *= *p*-value less than 0.050. TA=Tibialis Anterior; MG=Medial Gastrocnemius, N/A=Not applicable post-hoc analysis due to lack of statistical significance of the main effect.

S3 Table: Statistical result of onset latency as a function of group and side.

Latency (ms)		Paretic (Non-Dominant)		Non-Paretic		Group effect	Group Difference post-hoc analysis	Side effect
		Mean	S.E.	Mean	S.E.			
TA	Stroke	19.33	0.50	16.77	0.80	$F_{(2,27)}$=14.44, *p*<.001*, η^2=.52	Stroke vs. Age-Matched: *p*<0.001* Stroke vs. Young: *p*<0.001* Age-Matched vs. Young: *p*=0.956	$t_{(9)}$=2.82, *p*=0.20,*d*=0.35
	Age-Matched	15.91	0.49	--	--			
	Young	15.96	0.53	--	--			
MG	Stroke	17.89	1.03	16.74	0.56	$F_{(2,27)}$=1.72, *p*=.198	N/A	$t_{(9)}$=1.17, *p*=.272
	Age-Matched	16.63	0.69	--	--			
	Young	15.87	0.44	--	--			

Note: For group comparison, sMEP data of the paretic-side of the stroke group were compared with that of the non-dominant side of the healthy controls. Note: *= *p*-value less than 0.050. TA=Tibialis Anterior; MG=Medial Gastrocnemius, N/A=Not applicable post-hoc analysis due to lack of statistical significance of the main effect.
